# Multiple myeloma: challenges with deciding the optimal sequencing strategy

**DOI:** 10.3389/fphar.2023.1231720

**Published:** 2023-07-03

**Authors:** Anushka Walia, Alyson Haslam, Jordan Tuia, Vinay Prasad

**Affiliations:** ^1^ School of Medicine, University of California, San Francisco, San Francisco, CA, United States; ^2^ Department of Epidemiology and Biostatistics, University of California, San Francisco, San Francisco, CA, United States

**Keywords:** multiple myeloma, combination therapy, triplet regimen, doublet regimen, sequencing strategy

## Commentary

The therapeutic landscape for multiple myeloma (MM) has witnessed great advances over the past two decades, with more than 20 FDA-approved drugs currently available. The latest NCCN (Version 3.2023) guidelines recommend at least a triplet regimen as induction therapy for patients with newly diagnosed MM and adequate performance status. Quadruplet regimens are expected to be front-line in the near future, with trials evaluating combinations of daratumumab and bortezomib-lenalidomide-dexamethasone (VRd) currently underway. Yet despite the abundance of drug options for MM, the optimal sequencing strategy of these agents has not been determined. In this commentary, we contend that triplet and quadruplet combination therapies have not proven superiority over sequential therapy that starts with fewer upfront agents and reserves additional drugs for progression. Instead, randomized trials which have failed to adequately document or which have given suboptimal treatment at progression form the basis of the current dogma.

## PFS may not be a valid endpoint in studies evaluating combination over sequential therapy

Overall survival (OS) and health related quality of life (QoL) are the two important patient-centered endpoints in oncology. Historically in oncology, improvements in surrogates such as progression-free survival (PFS) or response rate have not been sufficient to prove superiority of combination over sequential therapy, when these benefits do not translate to improved OS or QoL. For instance, a 2003 phase III clinical trial comparing the combination of doxorubicin and paclitaxel vs. single agent doxorubicin or paclitaxel for metastatic breast cancer showed that combination therapy yielded superior overall response rates and longer time to treatment failure. (Sledge et al., 2003) Despite these benefits, the study authors rejected combination therapy on the basis of its failure to improve OS or QoL. Instead, the authors preferred single agent sequential therapy. The same logic has not been applied to multiple myeloma.

Multiple pivotal phase 3 clinical trials evaluating combination vs sequential therapy use primary endpoints of PFS. This raises several concerns. First, the validity of PFS as a surrogate for OS is questionable. In a recent analysis of 21 RCTs on newly diagnosed MM, the correlation (*R* ^2^) between PFS and OS was found to be just 0.65, which is “weak” according to standards published by the independent German Institute for Quality and Efficiency in Healthcare and suggests that improvements in PFS may not predict OS benefit. (Cliff and Rehman Mohyuddin, 2022; Etekal et al., 2023) While some MM RCTs such as SWOG 0777 and MAIA showed PFS and OS benefits with addition of bortezomib or daratumumab, respectively, to Rd at subsequent follow up, this was not the case in other trials. OCEAN and BELLINI demonstrated worse OS in experimental arms despite initial PFS gains (Cliff and Rehman Mohyuddin, 2022). The use of PFS as a primary endpoint is further complicated by the fact that progression has both clinical and biochemical definitions, which vary in prognostic value and are rarely reported separately in clinical trials (Villaruz and Socinski, 2013).

Second, even if PFS is a useful endpoint when establishing clinical benefit of a drug, this does not mean that it is equally valid to move a drug that has already proven PFS benefit up a treatment regimen. (Rajkumar et al., 2011). In trials evaluating triplet vs doublet therapy, patients in the doublet arm may receive the third drug at progression. A PFS benefit in the triplet arm is expected because each drug in the regimen has proven efficacy. Since patients may be able to achieve the same benefit by taking the third drug at a later time, the combination may not be superior to sequential therapy.

## Interpretation of OS benefit is confounded by lack of transparent reporting of post-protocol therapies

Transparent reporting of post-protocol therapies in randomized controlled trials (RCTs) is essential to compare the efficacies of combination and sequential therapies. Yet, one systematic review found that only 43.7% of 103 MM RCTs reported post-protocol therapies. (Mohyuddin et al., 2021a). Notably, the SWOG 0777 study, which established VRd over Rd as the standard of care in newly diagnosed MM, did not capture post-protocol therapies. According to the most recent follow-up, it is still unclear how many patients in SWOG 0777’s Rd arm received bortezomib at progression. Although the trial enrolled in the USA, where bortezomib was available, explicit knowledge of this information is key to ascertain the superiority of VRd over Rd with bortezomib made available as a salvage agent.

**FIGURE 1:**
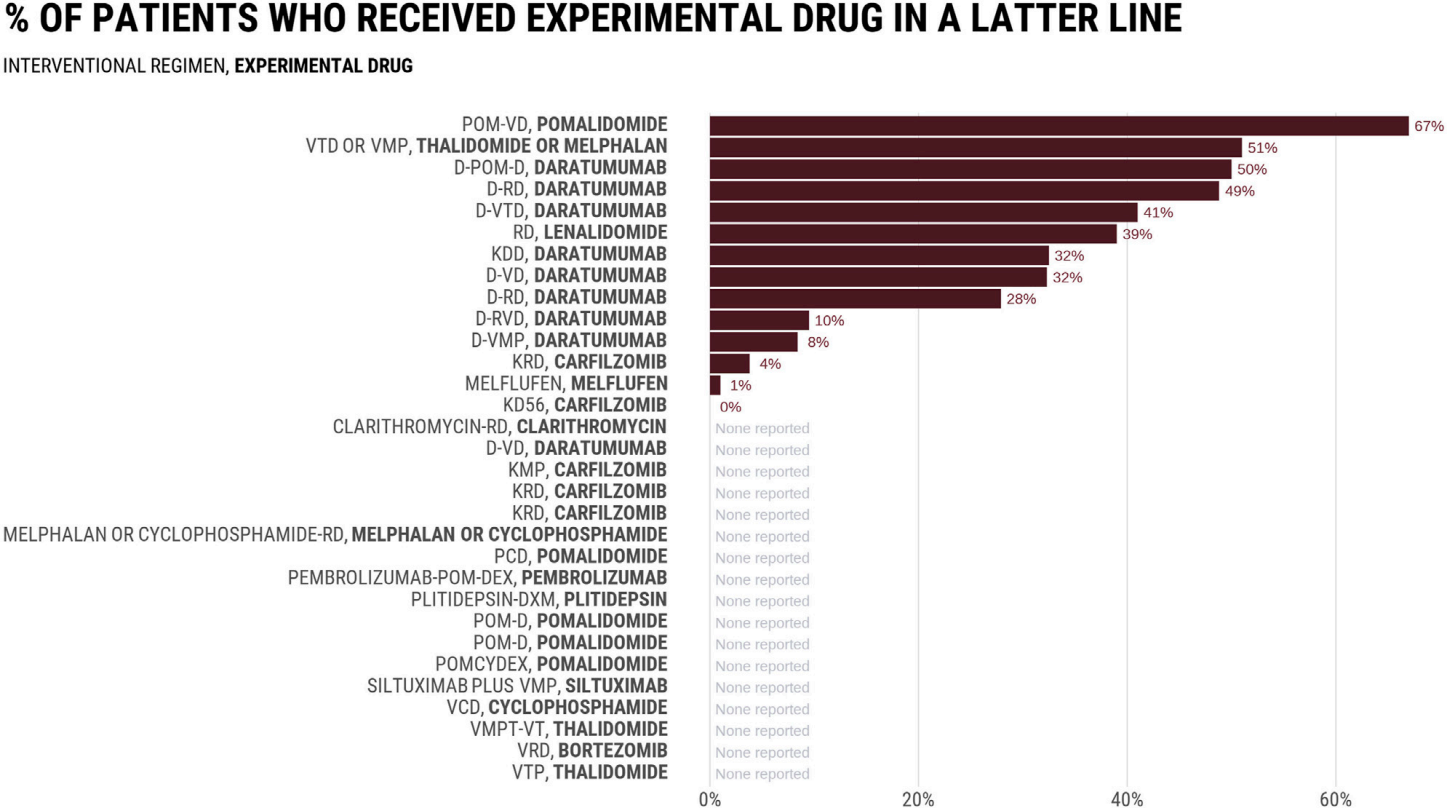
Percentage of patients who received experimental drug in a subsequent line.

Figure 1 reports the rates at which control groups received the experimental drug in a subsequent line in 32 RCTs evaluating combinations including daratumumab or carfilzomib, two drugs initially authorized by the FDA authorization in the salvage setting. Among 14 trials that reported subsequent therapies, the average rate was 29%, with numbers varying between 0% and 67%. In the MAIA study comparing daratumumab-Rd vs. Rd, 49% of patients in the Rd arm received daratumumab in a latter line. None of the three most common regimens received by control arm patients at progression (bortezomib; bortezomib-cyclophosphamide-dexamethasone, bortezomib-melphalan-prednisone) included daratumumab (Mohyuddin et al., 2021a). Rates were even lower in the ALCYONE study evaluating the addition of daratumumab to bortezomib-melphalan-prednisone (VMP), in which only 8.4% of patients in the VMP arm received a daratumumab-containing regimen as first line subsequent therapy. The PFS and OS improvements seen in experimental arms of such studies may not have existed if more patients in the control arm received the proper drug at progression. Thus, the question of whether combination therapy is superior to sequential remains unassessed. Trials have tested a trivial question of whether receiving the drug at some point during the cancer journey is better than never receiving it at all, but the question facing patients and doctors is when to give the drug to optimize outcomes.

Further complicating the interpretation of RCTs comparing MM regimens is the fact that control groups do not always reflect the current standard of care. In these studies, it is not possible to determine whether response rates reflect the superiority of the experimental treatment or the inferiority of the control treatment. A significant number of phase 3 MM RCTs have substandard control arms despite a superior regimen existing before or during the trial (Mohyuddin et al., 2021b). The MAIA, KEYNOTE-185 (pembrolizumab-Rd vs. Rd), and TOURMALINE-MM2 (ixazomib-Rd vs. Rd) trials for newly diagnosed MM illustrate a tension. Each of these trials had Rd control arms, despite results from SWOG 0777 emerging during enrollment. If the authors had accepted the superiority of VRd over Rd, which most likely did, then they were enrolling patients onto inferior control groups.

## Trial findings may not be generalizable to real world

Multiple myeloma has a median age of diagnosis of 70 years with a third of patients over 75. A significant proportion of MM patients are frail and have multiple comorbidities, both predictors of worse treatment outcomes. The benefits that triplet therapy shows in clinical trials may not translate to the real world, given that several combination therapies carry significant toxicities and trial criteria select patients with better performance status and fewer comorbidities.

Medhekar et al., 2022 evaluated outcomes among newly diagnosed non-transplanted MM patients treated with first line VRd in a real-world setting (Medhekar et al., 2022). They found median PFS to be 26.5 months, substantially lower than the 43 months reported in SWOG S0777’s VRd arm. Real world patients were also older (64% of patients over 65% vs. 38%) and more frail (48% vs. 21%) than those enrolled in the clinical trial.

The advantage of triplet over doublet therapy is less evident in the community setting. The community-based Phase IIIB UPFRONT trial compared bortezomib-dexamethasone (VD) with bortezomib-thalidomide-dexamethasone (VTD) and bortezomib-melphalan-prednisone (VMP) as induction therapy for newly diagnosed transplant ineligible MM. No differences in PFS between VD, VTD, and VMP were found, partly due to greater rates of adverse events and treatment discontinuation associated with triplet therapy.

Some real-world data suggests that initial doublet therapy with subsequent addition of a third drug if necessary can lead to successful outcomes. One retrospective study examined newly-diagnosed non-transplanted MM patients who received initial therapy with Rd and switched to a triplet therapy if VGPR was not achieved (Takezako et al., 2019). From VGPR rates of 32.3% after Rd alone and 69% after non-responders received an additional drug, the authors concluded that Rd is sufficient as initial therapy.

## Recommendations

When it comes to multiple myeloma, our overarching concern is that current paradigms will lead to more drugs given upfront to more patients, with greater attendant toxicity and cost, while patients and doctors remain fundamentally unsure if similar or superior overall survival and QOL could be achieved from careful, sequential use of these agents. Researchers have potential to ameliorate this situation.

First, transparent reporting of post-protocol therapies should be the standard in clinical trials comparing multidrug regimens and must be mandated by regulatory agencies. The majority of MM RCTs do not report this data and among those that do, the frequency of inadequate post-protocol care received by the control arm is alarming. Patients in control arms should have access to the experimental drug at minimum, if it has been authorized in the U.S. in a subsequent line.

Second, the use of PFS as a primary endpoint in trials investigating the addition of a drug to an existing therapeutic regimen should be avoided. A PFS benefit of a combination regimen is meaningless if the same OS or QoL can be achieved by providing the experimental drug in subsequent lines. While collecting OS data may require longer follow-up, this is not the case in all disease settings such as relapsed/refractory MM. For instance, for double refractory patients, median OS is 9 months, while the median PFS is 5 months—just a 4 months difference (Lee et al., 2013). Yet, the BELLINI and OCEAN trials used primary endpoints of PFS. If earlier results are desired, another possible option proposed by Cliff et al. is the intermediate endpoint, “PFS2,” which is equal to time until disease progression or death during the trial and after the first post-protocol therapy (Cliff and Rehman Mohyuddin, 2022). This can enable comparison between sequential and combination approaches until final OS results are achieved.

Third, QoL should be measured in all RCTs assessing combination therapies. QoL data has been absent from several practice-changing RCTs including SWOG 0777 (in which collecting QoL data is more important given bortezomib’s association with peripheral neuropathy). QoL measurements should be recorded throughout the length of the patient’s cancer journey, including throughout treatment and beyond progression (Haslam et al., 2020). Moreover, financial toxicity of multi-drug therapy should be accounted for in QoL scores and may be less apparent in the trial setting (Olivier et al., 2023).

Fourth, additional studies are necessary to determine how clinical trial findings translate to the real world, where significant differences in health status, financial burden, and treatment toxicity may exist. While some studies have highlighted discrepancies between real-world and trial outcomes in MM, they are limited by sample size and data on real-world treatment outcomes is currently sparse (Bertamini et al., 2022). Elsewhere, we have proposed the use of registry based pragmatic trials to achieve this aim, though other strategies may be complementary (Banerjee and Prasad, 2021).

Lastly, additional funding is required to perform trials specifically comparing “all at once” and sequential approaches to MM therapy. Existing RCTs were not designed for this purpose, given that they were funded by pharmaceutical companies and funding sources outside of industry are unfortunately limited. Lack of reporting of post-protocol care has further precluded any comparison of treatment strategies. Cooperative groups are well suited to champion this research agenda. Until we have this data, combination regimens with careful consideration of individual risk factors, treatment side effects, cost, and patient preferences can guide therapy in MM.

## Conclusion

Outcomes in MM have vastly improved in recent years as a result of novel anticancer agents and multidrug regimens. Despite the popularity of triplet induction therapy and a push towards quadruplets for MM, uncertainty remains. One treatment approach favors aggressive therapy with multi-drug combination regimens, the goal being to maximize initial response rates. A second approach favors sequential therapy that starts with fewer, less toxic agents and reserves a greater number of options for salvage lines.

Most clinical studies have shown that triplet or quadruplet regimens lead to deeper initial response rates than doublet therapy. However, it is not clear whether frontline doublet therapy followed by additional treatment upon progression would yield any less benefit. This question cannot be answered based on current trial data due to lack of reporting of post-protocol therapies or substandard post-protocol care. The survival and quality of life benefits of combination therapy are unclear for similar reasons. We do know that combination therapy leads to greater adverse effects, especially in older, frail patients that are typically excluded from clinical trials. Further data is necessary to justify the use of costly, toxic multi-drug regimens over sequential therapy that may prove just as favorable.
